# Nanoparticles Coated with Cell Membranes for Biomedical Applications

**DOI:** 10.3390/biology9110406

**Published:** 2020-11-18

**Authors:** Carla Jiménez-Jiménez, Miguel Manzano, María Vallet-Regí

**Affiliations:** 1 Department of Chemistry in Pharmaceutical Sciences, School of Pharmacy, Universidad Complutense de Madrid, UCM, Instituto Investigación Sanitaria Hospital 12 de Octubre, imas12, 28040 Madrid, Spain; carlaj05@ucm.es; 2 Networking Research Center on Bioengineering, Biomaterials and Nanomedicine (CIBER-BBN), 28029 Madrid, Spain

**Keywords:** nanomedicine, nanoparticles for drug delivery, cell membranes, coating technology

## Abstract

**Simple Summary:**

Nanomedicine has developed a new technology based on nanoparticles for drug delivery coated with different cell membranes. Although they were originally developed to increase their blood circulation time and stability though the use of red blood cell membranes, the versatility of this technology has extended to membranes from different cell types, such as white blood cells, platelets, cancer cells, mesenchymal stem cells, and beta cells, among others. Therefore, this cellular diversity and its unique properties, together with the possibility of using a wide range of nanoparticles and different drug dosage forms, has opened a new area for the manufacture of nanoparticles, with many potential applications in the clinic.

**Abstract:**

Nanoparticles designed for diagnosing and treating different diseases have impacted the scientific research in biomedicine, and are expected to revolutionize the clinic in the near future through a new area called nanomedicine. In the last few years, a new approach in this field has emerged: the use of cell membranes for coating nanoparticles in an attempt to mimic the ability of cells to interface and interact with physiological environments. Although such functions have been replicated through synthetic techniques, many research groups are now employing naturally derived cell membranes to coat different types of nanoparticles in an attempt to improve their performance for a wide range of applications. This review summarizes the literature on nanoparticles coated with cell membranes and, more importantly, aims at inspiring and encouraging new developments to this technology in the biomedical area.

## 1. Introduction

The recent development of nanotechnology has encouraged the application of new materials in various areas of science and engineering to satisfy certain requirements of society [1]. In this sense, various nanomaterials have been explored in medical research, giving rise to a new area in science, nanomedicine [2,3,4]. Among them, nanoparticles (NPs) for drug delivery offer significant advantages in terms of efficacy and safety in comparison with certain conventional therapies [5,6,7,8,9,10,11].

When nanoparticles are administered to the patient, they will encounter a highly complex and sometimes hostile environment developed to recognize and eliminate external elements. An example is what happens in the bloodstream, where certain proteins and cells of the reticuloendothelial system can prevent nanoparticles from reaching their target. Therefore, the nanoparticles should be designed for the appropriate interaction with the biological environment by reducing non-specific interactions and increasing the selective location in desired areas [12]. Traditionally, stealth nanoparticles have been functionalized with poly(ethylene glycol) (PEG), which promotes the creation of a layer of water molecules around the particles when dispersed in physiological media and increases their stability [13]. Stealth nanoparticles are capable of circulating unnoticed in the bloodstream, which increases their circulation time significantly. However, an increase in cases of immune responses to synthetic PEG is beginning to be observed, with a subsequent potential impact on the efficiency of these nanoformulations [14].

In the last few years, researchers have mimicked nature using cells for the design of bioinspired nanocarriers, particularly their membranes. The so-called cell-membrane-coated nanoparticles are composed of a synthetic nanoparticle surrounded by a layer of natural cell membrane. This cell membrane coating technology allows the design of biomimetic nanocarriers, whose surfaces replicate the complex properties of the source cells from which their membranes are derived [15]. Consequently, this new type of nanocarrier will present improved abilities to interface and interact with physiological environments, such as long circulation times and potential disease targeting capabilities [16,17]. This review summarizes the literature on nanoparticles coated with cell membranes, including some relevant examples of blood cell membranes employed to coat many different types of nanoparticles.

## 2. Cell Membrane

**Figure 1:**
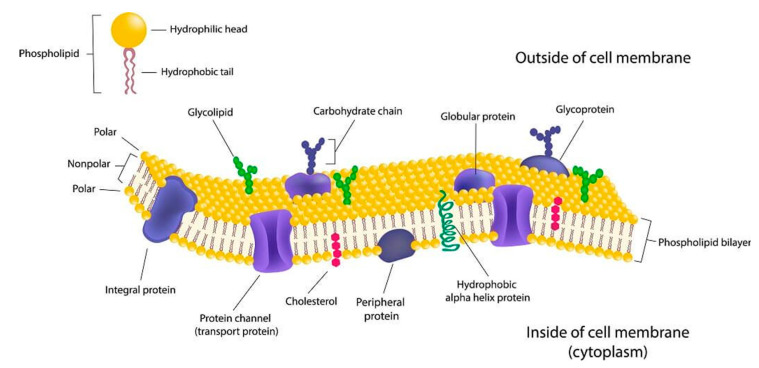
Cell membrane structure and its components (https://biologydictionary.net/cell-membrane/).

Cells interact with their surrounding environments through their membranes, which are composed of different proportions of lipids, proteins and carbohydrates (Figure 1). The cell membrane is a bilayer of lipids and other substances that delimit the entire cell, dividing the extracellular from the intracellular. Phospholipids are the major component of cell membranes, and present hydrophilic (polar) and hydrophobic (nonpolar) regions. The fatty acid “tails” of two phospholipid layers face each other. Therefore, the hydrophilic “heads”, which contain the phosphate moiety, face the environment and the cytoplasm inside the cell, where they form hydrogen bonds with the surrounding water molecules [18].

### 2.1. Composition

The plasma membrane of eukaryotic cells is made up of three types of lipids: sphingolipids, sterols, and glycerophospholipids. It is known that glycerophospholipids are those that provide the basic structure of the plasma membrane, thus being the predominant component in them. This type of lipid works as a second messenger or as a precursor in the generation of second messengers [19]. On the other hand, sphingolipids are found to a lesser extent in plasma membranes, even when they contribute, together with cholesterol (the sterol-type lipid present in cell membranes), to the heterogeneity of the organization of the cell membrane. They are responsible for regulating cellular processes such as growth, differentiation, cell apoptosis [20,21] or cytoskeletal reorganization [22]. It should be noted that many articles demonstrate how cholesterol acts as a modulator of the structural integrity, organization and fluidity of the membrane [23,24].

### 2.2. Interaction of Cells with the Surrounding Environment through Cell Membranes

The cell membrane has different functions. For example, the membrane is responsible for controlling the cell interaction with its environment. Each cell requires the presence of oxygen and other nutrients from the environment, so their membranes should guarantee the selective permeability of those vital elements. Membranes are also responsible for maintaining the balance of water with its environment and eliminating waste products from the cell. The membrane can carry out those tasks thanks to its permeability in a selective or differential way [25].

It also presents genetically unique cell recognition markers that are capable of providing mechanisms for a cell to recognize itself as “its own” versus “not its own” (foreign materials). This function is important for the immune system and for the defense of the organism or the maintenance of the cellular environment, since it regulates the materials that enter or leave the cell. The plasma membrane also serves as a boundary between the cellular cytoplasm and the external environment.

When there are interactions between the membrane and its microenvironment, the existence of large substances may require changes in the shape of the membrane and in the fusion of the membranes to be able to move things in or out of cells [25].

Cells need mass transport mechanisms with which they can move large particles or large numbers of smaller particles across the cell membrane. These mechanisms are as follows.

#### 2.2.1. Exocytosis

This process occurs when materials can be exported from the cell by fusing vesicles with the plasma membrane. This fact requires that the materials are packaged in the Golgi apparatus and the vesicles that have formed travel along the cytoskeleton to reach the plasma membrane. When the plasma membrane and vesicle membrane fuse, the contents of the vesicle are released to the cell [26].

#### 2.2.2. Endocytosis

This process occurs when substances enter the cell through membrane modifications. There are three endocytosis mechanisms by membrane modification: pinocytosis, receptor-mediated endocytosis, and phagocytosis [27].

- **Pinocytosis**

This process consists of the uptake of material from the extracellular space by invagination of the plasma membrane. Therefore, it is used to move fluids in or out of a cell. Whatever molecules are in the fluid will also move into the cell [28].

- **Receptor-mediated endocytosis**

Certain molecules (ligands) that the cell wishes to incorporate are recognized by specific receptors, located on the plasma membrane. Ligands bind to these receptors and these ligand–receptor complexes converge, thanks to the fluidity of the membrane. Then, these complexes will be endocyted. This is because the vesicles present on their cytosolic face a coating of characteristic proteins, in this case clathrin. The function of this protein, among others, would be to allow invagination to occur. The coated vesicle is then formed, and it will fuse with a set of vesicles called endosomes, where the endocytized molecules are classified and separated from the receptors [28].

- **Phagocytosis**

This process consists of the introduction of a solid into the intracellular medium, which can be a large molecule, a particle or a microorganism. This type of mechanism is carried out through phagocytes that absorb external pathogens, dead tissues or cells, surrounding them with their plasma membrane, remaining within the phagocytes. Afterwards, the pseudopods surround the absorbed particles, forming a vesicle [28].

## 3. Concept of Coating Nanoparticles with Cell Membranes

**Figure 2:**
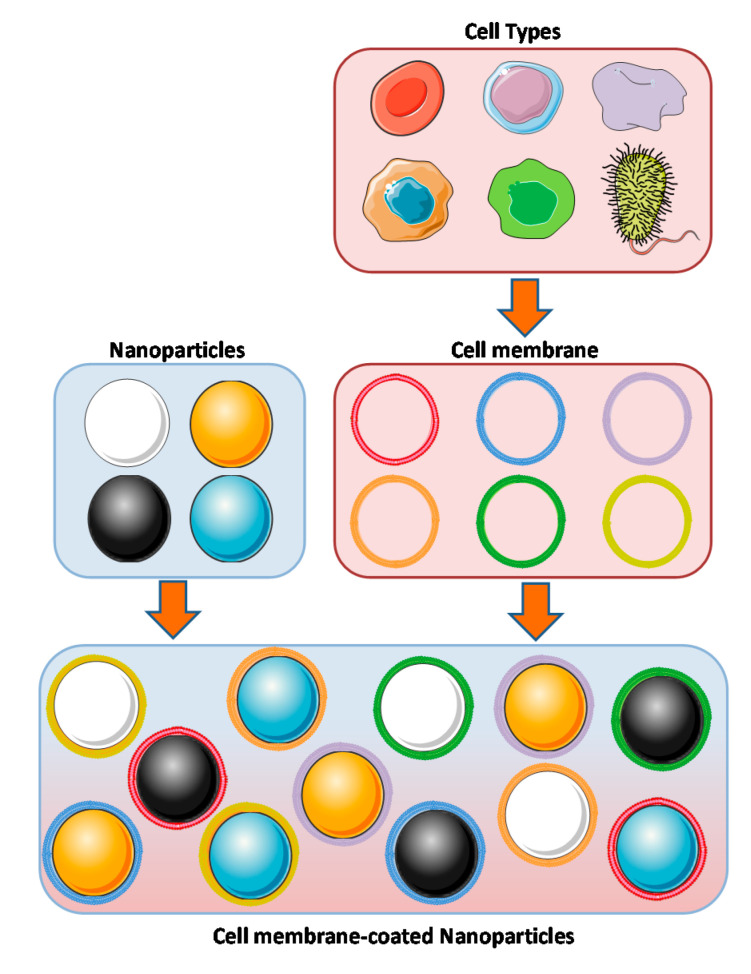
Cell membrane coated nanoparticles. Different types of cells have been used to coat over nanoparticles. Each type of cell membrane has different functions and properties depending on the required application.

In the last few years, the concept of coating nanoparticles with cell membranes was raised in view of the difficulty of developing synthetic strategies for the functionalization of nanoparticles. Therefore, nanotechnology sought inspiration from nature for the design of new nanotransporters, taking into account the interactions that nanoparticles would have with the physiological environment. Whether they act independently or form part of a multicellular organism, a cell comes into contact with a wide variety of other cells, proteins and/or extracellular matrices. That interaction takes place with such a great sensitivity and specificity through their lipid membranes, which inspired the development of biomimetic nanoparticles, combining the benefits of nanoparticles (core) and cell membranes (external surface). This new technology of coating with cell membranes allows the design of nanotransporters with surfaces that directly replicate the complicated functionalities required for a correct interaction with living tissues (Figure 2).

This concept was reported for the first time back in 2011, when the membrane from red blood cells was employed to coat poly(lactic-co-glycolic acid) (PLGA) nanoparticles [16]. Those cells were selected as membrane source because of their ability to circulate for extended periods of time, which is highly desirable for a nanocarrier. Since then, a wide range of different cell types have been employed for this technology, as described in the following sections.

Regarding the membrane coating methods, the easiest approach for coating already produced nanoparticles is based on subsequent extrusion procedures through porous membranes, mimicking those for the conventional synthesis of nanoliposomes. Sonication forces from ultrasounds have also been employed, showing better efficiency [29]. Additionally, microfluidic technology has also been employed to coat magnetic nanoparticles with better control thanks to the fine-tuning of the flow speed [30].

## 4. Principal Types of Cell Membranes Employed

### 4.1. Erythrocytes or Red Blood Cells (RBCs)

Erythrocytes are the most numerous cells in blood. Red Blood Cells (RBCs) can live up to 120 days in humans, and their role is to carry oxygen molecules all around the body [31]. They lack organelles, which facilitate the extraction and purification of the membrane, and they are a very promising cell type in the treatment of various diseases, since RBCs can be transporters of several active therapeutic molecules, as well as drugs, proteins and nucleic acids.

The first membrane employed to coat nanoparticles was the membrane derived from red blood cells. To date, this type of membrane coating is the most widely studied due to the ease of obtaining their membrane, which implies a rapid manufacturing. In addition, the polysaccharides on the surface of membranes from RBCs are very hydrophilic, which improves the stability of the NPs covered by this membrane [32].

Hu et al. were the first to extract the membranes from RBCs by hypotonic treatment in 2011 [16]. Later, oxygen-self-enriched red blood cells were designed, which had a high oxygen capacity and a prolonged circulation time [33]. Thanks to transmission electron microscopy (TEM), it was revealed that the RBCs membrane could coat NPs (65–340 nm in diameter) with proteins from the cell surface retained on the right side and also protect and stabilize the nanoparticles through lipid membranes and surface glucans [34].

On one hand, different studies have reported the existence of an electrostatic repulsion between the nucleus of nanoparticles and the extracellular side of the membranes, since both are negatively charged. This confirmed that the surface proteins present were oriented towards the outside of the membrane [34] and that the composition of the membrane proteins was maintained when the RBCs were used to coat the nanoparticles. Furthermore, these NPs have a longer half-life than uncoated NPs [16].

Other studies reported another type of application. As an example in one of them, the researchers observed that by coating the nanoparticles with iron oxide magnetic groups with RBCs membranes, they presented a higher contrast of MRI images and a higher heat conversion capacity [35]. Both properties are present in iron oxide [36].

On the other hand, this type of coating is used for the administration of drugs in various biomedical areas. The hypotonic method is the one used to load therapeutic agents [37] that allows the preservation of the immune and biophysical characteristics of the cell [38]. There are several studies where RBCs-NPs are applied in cancer. In an early article, doxorubicin (DOX) was loaded onto poly(lactic acid) (PLA) cores and then coated with a red blood cell membrane [39]. Drug loading was achieved by chemical conjugation and physical encapsulation. It was verified that the chemical conjugation of DOX with PLA allowed a high drug load and a more constant release over time. This fact was a consequence of the membrane coating layer serving as a diffusion barrier, which further slowed the kinetics of drug release.

Piao and Gao also demonstrated that CD47 (RBCs membrane marker) present in red blood cells inhibited phagocytosis by macrophages and protected gold particles from interaction with various compounds [40,41]. This demonstrated that the membranes derived from red blood cells are capable of improving the half-life of drugs in vivo. Another group developed nanoparticles coated by these membranes, which could suppress the inflammatory response induced by NPs and increase pro-inflammatory cytokines [42]. Recently, Wang et al. designed PLGA-NPs loaded with rapamycin and covered by a membrane of RBCs for the treatment of atherosclerosis. They demonstrated that the membrane from RBCs decreased macrophage-mediated phagocytosis in the blood and accentuated the accumulation of NPs, which leads to an improvement in the effect of the drug [43]. This technique is currently employed in the treatment of leukemia, atherosclerosis, and bacterial infections [44,45,46].

### 4.2. Leukocytes or White Blood Cells (WBCs)

Leukocytes are divided into different subtypes based on their different functions, such as macrophages, monocytes, granulocytes, lymphocytes, mast cells, neutrophils, eosinophils, and basophils. White blood cells (WBCs) are found scattered in different tissues and collaborate in different immune functions. They also present nuclei and very complex intracellular components, which means that it takes a long time to obtain their membranes. For this reason, the membrane derived from WBCs has recently been used to coat nanoparticles.

The membrane from WBCs is very useful since white blood cells have unique properties, such as their interaction with tumors or helping to fight infections [47]. This is due to the fact that when the body is infected or invaded, WBCs gather from the bloodstream to the injured site, causing inflammation and secretion of various cytokines [48]. It must be taken into account that WBCs have chemotactic properties and that the chronic inflammatory response is a main characteristic of tumor tissue. This fact makes many of the WBCs subtypes susceptible to tumor-directing chemoattractants.

Membrane-coated nanoparticles derived from WBCs are typically used in the delivery of anticancer drugs. As mentioned above, this is because WBCs can target sites where there is inflammation, which is one of the main characteristics in various stages of tumors and in tumor vasculature remodeling [49]. For example, nanoporous silicon NPs were coated with WBCs membranes through a purification process that consisted of a high density of sucrose [50]. These NPs were functionalized with positive (3-Aminopropyl)triethoxysilane (APTES), allowing the membrane coating to be stable. These coated NPs were also less subject to antibody opsonization (process by which a pathogen is marked for ingestion and destruction by a phagocyte) and serum protein adsorption compared to uncoated NPs. In another study, Zhang et al. showed that Interleukin 8 receptor (CXCR2) and Lymphocyte function-associated antigen 1 (LFA-1) played an important role in the localization of leukocytes in inflamed sites [51]. Inflammation and the ability to target tumors make leukocytes great candidates for the design of biomimetic NPs in photothermal therapy.

Monocytes are the precursors of macrophages and come from hematopoietic stem cells in the bone marrow. They tend to accumulate at tumor sites. Therefore, their presence is related to metastasis and tumor progression [52]. Monocytes are an important component of the body defense system, so their membrane is also used to create new delivery systems. Krishnamurthy et al. designed a monocyte membrane-coated-(PLGA-NPs) system loaded with DOX [53]. These coated NPs were found to maintain cell membrane heteroproteins. These membrane-coated NPs were also found to target tumor cells much more than uncoated NPs. On the other hand, Li et al. developed another drug delivery system, where NPs were coated with membranes derived from monocytes [54]. This coating prevented the NPs from being killed by the mononuclear phagocytes, since the membrane had the chemokine receptor that promotes tumor tropism. These results demonstrate that membrane-coated NPs derived from monocytes are of great interest in the delivery of drugs directed against tumors.

As mentioned above, macrophages are a type of immune cell that are derived from monocytes [55]. They are associated with cancer immunology as they can recognize and ingest tumor cells or any substance that does not present biomarkers on their surface [56]. Macrophage membranes have been used to make NPs that serve as drug delivery systems, which are widely used in cancer due to the proteins being present in their cell membrane [57,58].

In 2013, Tasciotti et al. designed nanoporous silicon NPs coated on these membranes to form leukocyte-like vectors. It was shown that there was a longer circulation time and accumulation in the tumor than when it was treated with uncoated NPs [50]. Membrane-coated NPs from macrophages were found to prevent uptake with the cell line from which the membrane was extracted where WBCs-coated NPs preferentially bound in inflamed endothelium. These nanoparticles were confirmed to be capable of penetrating certain layers of activated endothelium. Furthermore, these NPs improved transport compared to uncoated NPs. These NPs were loaded with DOX, and the presence of the drug did not affect their penetration capabilities into the endothelial layer to kill the deeper tumor cells. However, free drug or uncoated NPs killed endothelial cells without crossing the barrier [50].

In another report, Meng et al. designed Fe_3_O_4_-NPs covered by vesicles derived from macrophage membrane, where it was shown that these nanoparticles were biocompatible and that their membrane also presented immunoglobulin associated with membrane integrins (CD47), which leads to an immune evasion, and they were capable of attacking tumors [59]. In another study, gold NPs were coated with the membrane from macrophages, where it was observed that blood circulation time was improved, and they also accumulated at the tumor site [60]. A drug release was also proposed by Zhang et al., using the reduced pH of the tumor microenvironment, where it was observed that these NPs were highly biocompatible and that they also had the ability to localize tumors. Ultimately, this macrophage membrane lining has been shown to be of interest for the delivery of tumor-directed chemotherapy where there is a controlled release in response to stimuli from the tumor microenvironment [61].

In addition to drug delivery applications, another leukocyte membrane derived from lymphocytes has been studied. These are classified into T cells, B cells and natural killer cells (NK) depending on the moment of their appearance, the surface molecules and the functions that each one presents [62]. The cytotoxic T lymphocyte membrane has been used for coating NPs. These are able to precisely recognize the antigen of tumor cells and consequently activate the antitumor immune response, making them good candidates for the creation of a biomimetic administration technique [62]. Low-dose local electromagnetic irradiation was used together with membrane-coated NPs from these cytotoxic T lymphocytes in the treatment of tumors, observing that the circulation time was longer and that the ability to target the tumor was improved. For the treatment of the gastric tumor, PLGA-NPs loaded with paclitaxel (PTX) and covered by T cell membranes belonging to human blood samples were used. The presence of T cell markers was verified in these membranes, highlighting LFA-1 and CD3z (CD3 zeta chain of T cell surface glycoprotein). This fact indicated that these NPs had immune evasion capacity [62]. Furthermore, an in vivo experiment demonstrated that when mice were treated with these NPs and low-dose local irradiation, localization of these NPs improved the localization of these NPs at the tumor site compared to non-irradiated mice. An increase in the expression level of Intercellular Adhesion Molecule 1 (ICAM-1), which is a ligand for LFA-1, was also demonstrated, observing that this adhesion molecule was upregulated in tumor vessels after applying irradiation [62]. During this process, the interaction between LFA-1 and ICAM-1 is crucial. Moreover, the biological response to this irradiation could enhance the binding interaction between the tumor and the membrane-coated NPs from T lymphocytes, improving antitumor efficacy.

Recently, PLGA-NPs loaded with trametinib and coated with membrane from T cell hybridoma have also been used for the treatment of melanoma [63]. These NPs were found to be very stable and drug release was dependent on the amount of membrane used due to the presence of a melanoma-specific anti-gp100/HLA-A2 T-cell receptor (TCR) that promotes binding kinetics and cell uptake. Furthermore, these NPs were endocytised to a greater extent by tumor cells compared to uncoated NPs, which led to significant tumor killing in vitro. Therefore, these nanoparticles could be used in therapy associated with melanoma [63].

The membrane from NK cells has also been used for coating NPs. These nanoparticles, formed by fusogenic liposomes infused into NK cell membranes, were loaded with DOX for targeted therapy of cancer. Tumor cells endocytosed these coated NPs before DOX was released, thus allowing the drug to be released into cells [64]. To date, membrane extraction from B cells for coating NPs has not been performed.

The neutrophil membrane has also been used in the coating of NPs for the treatment of tumors. For example, Kang proposed membrane-coated NPs derived from neutrophils and loaded with carfilzomib (CFZ), which were capable of targeting tumor cells and inhibiting the premetastatic zone [65].

On the other hand, NPs coated with WBCs membrane can be used for the elimination of endotoxins and for the reduction of inflammation. In one study, PLGA-NPs were coated by macrophage-derived membranes to phagocytize lipopolysaccharide (LPS) and pro-inflammatory cytokines in the treatment of sepsis [66]. It was found that the coating did not affect the functionality of the cytokine-binding receptors and that, in fact, the coated-NPs cleared the cytokines in a concentration-dependent manner. It was also found that these NPs retained important surface proteins in binding with LPS and that the neutralization of this lipopolysaccharide is a characteristic of the macrophage membrane [66]. Ultimately, the biological functions that WBCs present, such as targeting tumor cells, immune evasion, elimination of endotoxins, and reduction in inflammation make them very attractive candidates in the design of new nanocarriers in the treatment for different pathologies.

### 4.3. Platelets

Platelets or thrombocytes are fragments that lack a nucleus of megakaryocytes and originate in the bone marrow. Their main function is to maintain hemostasis, since they are recruited where there is a vascular lesion, initiating the healing process through the formation of clots. The platelet-derived membrane is used because the expression of its surface marker is maintained, as well as the antigens and proteins present in the original platelets. Platelet membrane-coated NPs have many biomedical applications, such as the treatment of immune thrombocytopenia [67], the administration of drugs [68] and the treatment of cancer [69].

In 2015, PLGA-NPs coated with membranes derived from platelets were used. These coated nanoparticles were observed to keep the expression of their surface marker and the functionality of the immunomodulatory and binding proteins. These coated nanoparticles, through membrane glycoprotein receptors, were found to bind to human type IV collagen. This fact is one of the main functions of platelets. Macrophage uptake was found to decrease compared to uncoated NPs. Apart from that, they were also found to be stable and biocompatible nanomaterials. In an in vivo analysis, these NPs were loaded with docetaxel to treat restenosis following angioplasty. In observed mice, these NPs were shown in these experiments to be guided into the damaged vasculature and to remain stable for 5 days. These platelet-membrane-coated docetaxel-loaded NPs released the drug that significantly reduced the intimal layer of the arterial wall compared to free drug or uncoated NPs [17].

In cancer, these platelet-derived membrane-coated systems are also used for drug delivery. In the platelet membrane, the protein P-selectin that specifically binds to CD44, which is induced in tumor cells, is overexpressed [70]. This fact helps the localization of the tumor and subsequent drug release. In one study, NPs were loaded with DOX and coated with platelet membranes [71]. In vitro studies demonstrated that the drug release was higher at acidic pH. Furthermore, these DOX-loaded NPs were functionalized with the apoptosis-inducing ligand (TRAIL). It was found to be a very powerful system against a breast cancer cell line. In vivo analysis showed that this coating was able to control tumor growth and significantly decrease the number of metastatic nodules. Therefore, this nanosystem is beneficial for targeted drug delivery, causing both extrinsic and intrinsic apoptosis [71].

Xu et al. designed a nanosystem of PLGA-NPs loaded with verteporfin and covered with a membrane derived from platelets. These NPs join tumor cells in contact with reactive oxygen species, helping to fight the tumor without causing damage to the skin [69]. In a different approach, Jing et al. developed NPs loaded with DOX and melanin and coated with platelet membranes that were modified with the arginyl-glycyl-aspartic peptide [72]. This modification leads these platelet membrane-coated NPs to have the ability of immunological evasion and be able to direct this peptide towards the αvβ3 integrin, thus being able to combat tumor vasculature [72].

Another example was the development of NPs loaded with bortezomib and coated by platelet membranes for the treatment of multiple myeloma [73]. The drug release killed tumor cells in a dose-dependent manner. This fact did not occur with non-functionalized NPs. These nanoparticles were then functionalized with alendronate to prevent bone loss and thus be able to accumulate NPs near the tumor site. This formulation facilitated binding to calcium-rich hydroxypatite in femur and bone marrow tissues. Tissue plasminogen (tPA) was also added to these NPs to assist in the elimination of clots that arise in the treatment of multiple myeloma. tPA was shown to retain its function and be more stable over time compared to free tPA. Furthermore, treatment with these NPs decreased thrombosis. However, it was observed that both NPs, with or without tPA, improved survival and induced more apoptosis in bone marrow in a mouse model [73].

### 4.4. Cancer Cells

Membrane extraction from cancer cells is one of the most important sources in nanomedicine because they show specific targeting to the source cell [74] and manifest a homologous adhesion mechanism to other cancer cells [75,76,77]. Additionally, this membrane is robust, easy to culture and can be easily removed. Unlike other cell types, cancer cells exhibit unique properties, such as a self-targeting ability that can be used for drug delivery, replicative immortality, angiogenesis, and activating invasion and metastasis [78]. NPs coated with this membrane are stable and they can transport antigens from the tumor membrane to tumor sites, improving cancer efficiency [74]. Today, this technique is widely used to study targeted drug delivery in cancer treatment [79,80].

Free-chemotherapy drugs lack effective abilities to target tumors. Therefore, the coating of drugs with the membranes of cancer cells can contribute to improving the efficiency of the damage to tumors. This sense, Li et al. designed poly(caprolactone)-NPs loaded with PTX and covered with a cancer cell membrane, which were used for chemotherapy targeting the 4T1 tumor. In these NPs, it was shown that the membranes maintained the expression of the 4T1 surface antigens and could accumulate at the sites of tumors or metastases. Furthermore, these coated NPs decreased tumor growth in mice compared to the control group or the group treated with uncoated NPs. Treatment with these NPs was shown to reduce metastasis [81]. Therefore, these NPs were confirmed to be a good drug delivery system.

In a different study, membrane-coated PLGA-NPs were developed from melanoma cells. These coated NPs were found to be stable over time. Furthermore, a good colocalization between the membrane and the nucleus of the dendritic cells from the bone marrow was demonstrated, confirming the existence of a homotypic union. These cancer-membrane-coated NPs were compared to RBCs-NPs and uncoated NPs. Tumor membrane-coated NPs were shown to have greater binding to parent cancer cells compared to the other two types of NP [74].

In another report, the concept of homotypic orientation for drug delivery was extended. Magnetic Fe_3_O_4_-NPs were loaded with DOX by electrostatic interaction and coated with membrane from various tumor cell lines. These coated NPs were found to maintain the membrane markers but lost numerous intracellular components, in addition to good internalization by cancer cells. These NPs were also observed to release the DOX drug in a pH-dependent manner. Once again, the homotypic and heterotypic union between NPs covered by membranes of different cell types was analyzed. It was found that there was a better internalization of these NPs by the cell line from which this membrane coating was derived, a fact that did not occur between different cell lines. In vivo, membrane-coated NPs from H22 cancer cells were shown to be better internalized by H22 tumors in mice, including even if the mice were another tumor type from another cell line. Consequently, they concluded that membrane-coated NPs from a tumor cell line only targeted tumors that had the same type of cells. By taking advantage of the core material, a magnetic field could also be applied to guide the nanoparticles to the tumor site and further improve efficiency [82].

On the other hand, tumors exist in a hypoxic environment, which leads to drug resistance. Recently, PLGA-NPs loaded with hemoglobin and DOX and coated with a tumor membrane have been designed to avoid chemoresistance caused by tumor hypoxia. Again, it was found that there was a homotypic union between the membrane and the cell line from which it came. It should be mentioned that these NPs were more toxic than free drug or uncoated but drug-loaded NPs. After that, an experiment was carried out under hypoxic conditions, where it was observed that the application of these NPs triggered the negative regulation of HIF-1α and P-glycoprotein, which are responsible for the release of drugs. Thanks to this, these coated NPs released a higher amount of DOX in tumor cells compared to uncoated NPs or non-oxygenated NPs. In vivo experiments, this same fact occurred. In consequence, it was confirmed that there is a synergy between the coating of the tumor membrane and the oxygen load since it increased the release of DOX in the tumor cells of mice, improving the survival of these mice [83].

These nanocarriers are also used as cancer vaccines [84]. For example, Zhang et al. developed NPs with an adjuvant layer of the TLR4 monophosphorylipid A (MPLA) agonist and membrane-coated cancer cells, which could be used for vaccination. With these NPs, they wanted to study the maturation of dendritic cells and the release of tumor antigens. Only with these NPs were they able to proof the existence of effective dendritic cell maturation. This group then performed an in vivo model where they combined dendritic cells with pmel-1 transgenic murine splenocytes specific to a particular tumor antigen. With this experiment, they confirmed the existence of a significant accumulation of T cells around dendritic cells and cancer cell membrane treated with the MPLA-coated NPs and a significant induction of interferon gamma (IFNγ) secretion [74]. Li et al. designed, in a different study, multiantigenic NPs coated on cancer membranes, which presented many cancer-associated antigens and were administered as cancer vaccines. In vivo, its immune stimulation capacity was analyzed, where it was discovered that prophylactic vaccination with nanovaccine that presented these multiantigenic and coated NPs showed the antigen more efficiently by activating dendritic cells, thus improving the effectiveness of tumor prevention. Furthermore, the survival of the mice treated with these NPs improved compared to the rest of the groups [85].

In summary, cancer-cell-derived membranes manifest many tumor-associated antigens to stimulate tumor-specific immune responses. Consequently, it is confirmed that adjuvant encapsulation is an effective technique to stimulate immunotherapy against cancer.

### 4.5. Mesenchymal Stem Cells

Mesenchymal stem cells (MSCs) or stromal stem cells are multipotential progenitor cells that possess the capacity for self-renewal. They are used in various clinical applications, highlighting regenerative medicine. They are also used in reducing side effects after the use of chemotherapy or in the treatment of autoimmune diseases. In nanomedicine, these membranes are used for coating nanoparticles because they are easy-to-work cells since they can be quickly isolated, as well as having a lot of information for large-scale cultivation. MSCs possess various properties, such as tumortropism, which have been exploited to provide useful therapeutic loading [86,87]. The use of the membrane of these cells to coat NPs has made it possible to manufacture nanocarriers with similar orientation functionality. In recent years, the role of these cells in tumor development and metastasis, as well as the targeted delivery of anticancer drugs, has been studied. He et al. designed membrane-coated DOX and gelatin nanogels from bone-marrow-derived mesenchymal stem cells, which showed stability in the long-term. In addition, they exhibited tumor-pH-sensitive drug release behavior and high drug loading capacity [88]. These nanogels were less toxic than the naked NPs or the free drug. They presented high ability to attack tumors and increased accumulation at the tumor site. Another similar drug delivery system, which was coated with PLGA and DOX, exhibited antitumor and tumor localization characteristics [89]. On the other hand, stem cell membranes have been used to mask NPs, improving the efficiency of deep tissue photodynamic therapy tumoral treatment [90]. Briefly, the MSCs membrane has shown a high capacity for drug delivery and tumor targeting. Therefore, is believed that it will improve the photothermal therapy by coating agents with a high heat conversion rate and achieve good results in excision of tumors.

In regenerative medicine, nanoparticles coated with this type of membrane were employed as a replacement for living stem cells, reducing the tumorigenicity and immunogenicity of living cells but without modifying the interactions of stem cells with damaged cells [91].

Adipose-derived mesenchymal item cell membranes were used to coat iron oxide nanoparticles for MRI applications [92]. Recently, superparamagnetic iron oxide nanoparticles, which were loaded with doxorubicin and coated with mesenchymal stem cell membranes, have been designed to explore their effect on colon tumors. These coated NPs were shown to improve cellular uptake efficiency and antitumoral effects, as well as reducing the immune system response. Therefore, it was confirmed that these NPs can be a targeted nanocarrier in the treatment of colon cancer [93]. Another group developed nanocarriers coated with stem cell membranes functionalized to express CXCR4 by improving penetration of the endothelial barrier and decreasing uptake by human and murine macrophages [94]. Today, this type of coating is widely used in different therapeutic applications.

### 4.6. Bacteria

Bacteria are microorganisms that lack organelles and nuclei [95]. Membranes from pathogens have also been extracted to create biomimetic materials to prevent or treat diseases. For example, NPs were coated by the *E. Coli* membrane for drug delivery in neutrophils [96]. In a process of inflammation, neutrophils are attracted to the inflamed area. This coating facilitated the endocytosis of these NPs by neutrophils compared to non-functionalized NPs, maintaining biocompatibility. They also confirmed that neutrophils that had engulfed these NPs migrated to bacteria that were immobilized on the gel [96].

A different group designed PLGA-NPs coated by extracellular vesicles from *S. aureus*. They observed that these NPs targeted infected macrophages and sites infected by these bacteria. These NPs were shown to accumulate at sites that had a higher bacterial load in infected mice compared to the non-infected group. Afterwards, these NPs were loaded with antibiotics, which decreased the bacterial load [97].

## 5. Other Cell Membranes Used for Coating NPs

### 5.1. Beta Cells

Beta cells are responsible for insulin secretion and constitute 70% of pancreatic cells. Therefore, the membrane extraction of these cells is of interest for the study of diabetes mellitus, which affects more than 425 million people in the world. In a study, polycaprolactone nanofibers coated by the membrane of these cells were cultured with beta cells, which showed that these coated fibers maintained the membrane proteins. Furthermore, the presence of these nanofibers increased the survival, proliferation, and insulin production of these cells compared to the culture that had been treated with uncoated nanofibers [98]. This technique can be used for any cell that needs input signals when there is cell–cell contact for proper growth and survival.

### 5.2. Endothelial Cells

These cells are necessary for the maintenance of a healthy vasculature. For example, different types of NPs were developed, covered with endothelial cell membrane and incubated with HUVEC cells. The researchers found that these NPs were very well internalized. Vesicles containing these NPs were forced to release by starvation. Finally, they observed that charged and coated Fe_3_O_4_-NPs could be directed to the desired location using a magnetic field [99]. Therefore, this technique could be used for the design of drug delivery nanosystems in various applications.

### 5.3. Fibroblasts

The extraction of membrane from fibroblasts is of interest, since they can be used in different pathologies. For example, it is used in the treatment of diabetes where a PLGA diaphragm was covered by a fibroblast membrane in order to differentiate pancreatic stem cells and generate cells that produce insulin. This fact contributes to the existence of an affinity for the PLGA membrane while, at the same time, it is possible to examine the differentiation of these stem cells, important in the development of artificial islets for the treatment of diabetes [100].

On the other hand, fibroblasts promote the angiogenesis, metastasis, and proliferation of tumor cells [101]. Semiconductor polymeric NPs were coated with membranes from fibroblasts, allowing these NPs to target tumor-associated fibroblasts. In an in vivo photodynamic therapy experiment, a 4T1 xenograft was used. First, the mice were treated with these NPs and 48 h after this intervention, the mice were irradiated. Mice that had been challenged with these coated NPs were found to have a higher temperature and more tissue damage than the control group [102]. This fact led to complete photodynamic therapy-mediated tumor elimination.

## 6. Conclusions and Future Perspectives

Different designs and applications of nanoparticles coated with different cell membranes in nanomedicine have been reviewed here. These nanosystems were originally developed to increase their circulation time, so membranes from red blood cells were initially explored, since those cells are known to circulate in blood for long periods of time. The versatility of this technology has fuelled its use with cell membranes from different types of cells, such as white blood cells, platelets, cancer cells, mesenchymal stem cells and beta cells, among others. Therefore, this cellular diversity and unique properties, together with the possibility of using a wide range of nanoparticles and different drug dosage forms, has opened a new area for manufacturing nanocarriers with many applications.

It is worth mentioning that cell membranes show different properties, such as controlling biological interaction, immune escape, and homologous targeting. One of the most interesting aspects of the technology presented here is that those cell membrane properties are maintained after coating the nanoparticles. Therefore, the potential applications of these nanocarriers are almost unlimited and remain to be explored.

In the clinical field, coating nanoparticles with cell membranes has raised great interest regarding the potential treatment of diseases such as bacterial infections and cancer due to the immunological modulation from membranes. In fact, the administration of these coated nanoparticles has been shown to promote an increase in antigen-specific immunity. This coating technology is also used for developing nanovaccines that might promote extensive immunity to tackle the required targets. In the future, membrane-coated particles could be used to stop the autoimmunity of a specific form of antigen.

In summary, this technology has great advantages and cellular diversity, which can cover different approaches in medicine. However, it has not yet been fully applied in the clinic. In the future, we believe that nanocarriers will be developed that can be applied in medicine helping human health.
